# A methodology for refining AR-DRG

**DOI:** 10.1186/1472-6963-11-S1-A14

**Published:** 2011-10-19

**Authors:** CW Aisbett

**Affiliations:** 1 Laeta Pty Ltd, Randwick, New South Wales, 2031, Australia; 2 Casemix Unit, Health Service Executive, Palmerston, Dublin, Ireland

## Introduction

Previous reviews of AR-DRG, such as that by Aisbett, Wiley et al (2007), have shown prior versions of AR-DRG to be among the world’s best in practice, and that further major improvements in grouper performance are unlikely to have occurred. More recent work, such as that by Aisbett, Aisbett, Sutch et al (2008), has shown that hospitals dealing with (age) restricted sub-populations may be disadvantaged by funding mechanisms based on AR-DRG.

The understanding here is that DRG systems rely on population-sampling assumptions (as well as matching on influential variables and mathematical modeling) to reduce the risk of biased comparisons of health services. Aisbett’s methodology can be used to identify sets of medical conditions (and procedures) that are associated particularly with increased risk of bias. The research findings also encourage development of AR-DRG along the lines of age-dependent complication levels, so it is appropriate to examine how this knowledge can be implemented to achieve effective refinement.

The ultimate aim of this work is to make changes to the current grouper that will lead to better performance as evaluated under the criteria used in the two publications referred to above.

## Methods

National and international studies have provided lists of medical codes associated with complication of care. These data can often be sourced for use in research. For example, one outcome of the work in The Information Centre, Casemix Service, National Health Service UK (2007), is the tabulations of secondary diagnosis codes associated with extra-care requirements in paediatrics. These data are directly useful, as demonstrated in Aisbett et al (2008), or as stimulus material for subject matter experts.

Large Australian data sets also allow for the further exploitation of the methods outlined in Aisbett et al (2008). These were based on the examination of resource relativities as exhibited across the health system, and within particular subsets of the system. Aisbett's Generalized Least Squares (GLS) method identifies when the assumption that there are no material interactions between AR-DRG resource relativities and episode-of-care sub-population fails. This knowledge can then be used in conjunction with code frequency-based partitions of resource use data to identify codes that have age-specific complicating effects.

The process of evaluation of a code’s complication and comorbidity level (CCL) (or the impact of a group of codes) can be outlined as follows:

1. Divide the AR-DRGs into a High and a Low set according to the (relative) frequency of the code as a secondary diagnosis in that particular AR-DRG in the whole collection.

2. Divide the collection into subsets according to demographic/health service variables of interest.

3. Conduct Aisbett’s GLS analysis using only the AR-DRGs in each set (High and Low), but use the same demographic/health service variable breakdowns as episode-of-care subsets (sub-populations).

4. Identify whether the resource relativities assumption fails in the High frequency set.

5. For each sub-population, identify whether the Casemix-adjusted cost per episode differs for the High and Low frequency partition.

6. Analyse the outputs above to see if there are interaction effects.

## Results

Detailed results are available in Chapter 4 of the publication available at the following web address: Costing Kid’s Care [http://www.pc.gov.au/__data/assets/pdf_file/0017/90611/sub021-attachment.pdf]

In essence, groups of codes associated with the distortion of AR-DRG resource relativities may be identified even when individual codes have a low frequency of occurrence.

## Conclusions

The approach developed in this research may be applied to a wide range of grouper development initiatives.

